# Atm inhibition decreases lens opacity in a rat model of galactose-induced cataract

**DOI:** 10.1371/journal.pone.0274735

**Published:** 2022-09-23

**Authors:** Masaya Nagaya, Fumito Kanada, Masaru Takashima, Yoshihiro Takamura, Masaru Inatani, Masaya Oki

**Affiliations:** 1 Department of Industrial Creation Engineering, Graduate School of Engineering, University of Fukui, Fukui, Japan; 2 Faculty of Medical Sciences, Department of Ophthalmology, University of Fukui, Fukui, Japan; 3 Life Science Innovation Center, University of Fukui, Fukui, Japan; National Center for Toxicological Research, UNITED STATES

## Abstract

Cataract causes vision loss and blindness due to formation of opacities of the lens. The regulatory mechanisms of cataract formation and progression remain unclear, and no effective drug treatments are clinically available. In the present study, we tested the effect of ataxia telangiectasia mutated (Atm) inhibitors using an *ex vivo* model in which rat lenses were cultured in galactose-containing medium to induce opacity formation. After lens opacities were induced by galactose, the lenses were further incubated with the Atm inhibitors AZD0156 or KU55933, which decreased lens opacity. Subsequently, we used microarray analysis to investigate the underlying molecular mechanisms of action, and extracted genes that were upregulated by galactose-induced opacity, but not by inhibitor treatment. Quantitative measurement of mRNA levels and subsequent STRING analysis revealed that a functional network consisting primarily of actin family and actin-binding proteins was upregulated by galactose treatment and downregulated by both Atm inhibitors. In particular, *Acta2* is a known marker of epithelial-mesenchymal transition (EMT) in epithelial cells, and other genes connected in this functional network (*Actn1*, *Tagln*, *Thbs1*, and *Angptl4*) also suggested involvement of EMT. Abnormal differentiation of lens epithelial cells via EMT could contribute to formation of opacities; therefore, suppression of these genes by Atm inhibition is a potential therapeutic target for reducing opacities and alleviating cataract-related visual impairment.

## Introduction

Cataracts cause visual impairment due to lens opacity, and are the leading cause of blindness worldwide [1]. Cataracts are caused by a variety of factors, including aging and ultraviolet rays, but diabetes accelerates the onset of cataracts and increases the risk of developing cataracts by 2–5-fold [2,3]. Currently, the only clinical intervention for cataracts is to surgically remove the lens opacity and implant an intraocular lens; however, this treatment is not available in some facilities of developing countries, and it carries a palpable risk of side effects [4,5]. For this reason, research efforts have aimed to prevent and treat cataracts using pharmacological approaches [6,7], but no effective therapeutic agents are currently available in the clinic.

While age-related cataracts tend to cause opacities in the lens nucleus, diabetes-related cataracts commonly cause opacities in the lens cortex [8,9]. *In vivo* models, such as the streptozotocin diabetic rat model and galactose-induced cataracts are commonly used as diabetic cataract models [10,11]. *Ex vivo* models, in which cataracts are induced by culturing lenses in galactose-containing medium, are also commonly used to simulate diabetes-induced cataracts [12,13]. Galactose-induced cataracts and diabetic cataracts share a common mechanism [9], and the galactose *ex vivo* model can rapidly and stably induce lens opacities, so is useful for drug screening. The mechanisms underlying formation of sugar-induced cataracts include production of membrane-impermeable sugar alcohols [14], oxidative stress, and accumulation of advanced glycation end products [15], and the disease is thought to originate via a complex combination of these factors [16].

In addition, increased lens epithelial cell (LEC) apoptosis due to oxidative stress and high sugar levels has been implicated in the development of diabetic cataracts in both humans and animal models [17,18]. Preventing the accumulation of sugar alcohols by inhibiting aldose reductase (AR) decreases LEC apoptosis and prevents cataract onset [18,19], and LEC apoptosis is considered to be an important event in the initiation of cataracts. In addition, epithelial-mesenchymal transition (EMT), in which LECs differentiate into mesenchymal cells, is implicated in cataracts via AR [20,21]; indeed, recent studies report that EMT may contribute to formation of diabetic cataracts [22–24]. The role of EMT in lens posterior capsular opacification, which primarily presents as a complication of cataract surgery, is well-established [25], but the potential role of EMT in diabetic cataract remains untested. Maintaining LEC homeostasis is important in protecting lens fiber cells within the lens [26]. Loss of LEC integrity by apoptosis or abnormal cell phenotypes could contribute to lens opacity, but further investigation is needed to elucidate the detailed regulatory mechanisms.

Previously, we reported that Polo like kinase 3 (Plk3), which is involved in cell cycle progression and apoptosis, and ataxia telangiectasia mutated (Atm), which is upstream of Plk3, are involved in galactose-induced cataracts [13]. GW843682X, a Plk3 inhibitor, and KU55933 an Atm inhibitor, prevent galactose induction of cataracts *ex vivo*. Atm is one of the most important regulators of the double-strand break DNA damage response, and activates downstream regulators of various cascades such as DNA repair, cell cycle arrest, and cell death [27,28]. ATM is recruited to DNA double-strand breaks, where it activates various downstream signals by phosphorylating proteins such as p53 and chk2 [27]. Although preliminary reports that have evaluated the relationship between Atm and cataracts, as well as the direct relationship between DNA damage and cataracts, it is well-established that use of ionizing radiation to induce DNA damage alone results in cataract formation [29]. In addition, age-related cataracts show increased levels of DNA damage [30]. Because DNA damage is caused by reactive oxygen species produced when the cellular redox state is compromised, the DNA damage response could also contribute to opacity formation in diabetic cataracts.

Previous studies have demonstrated that Plk3 and Atm prevent rat galactose-induced cataracts, but did not evaluate whether these inhibitors could reverse the opacity once formed. In the present study, we examined the effects of Plk3 and Atm inhibitors on galactose-induced cataracts that had already formed, revealing that Atm inhibitors reduced lens opacity. Gene expression in lenses ± galactose and inhibitors was investigated by microarray analysis, which identified that genes related to the cytoskeleton were upregulated by cataract formation, which was suppressed by Atm inhibitors. These findings further advance our understanding of the mechanisms of diabetic cataract progression and suggest that targeting Atm and downstream genes is a putative approach for alleviating lens opacity.

## Materials and methods

### Animals

Six-week-old male Sprague-Dawley rats used for experiments were purchased from Sankyo Laboratory Service. All experiments were approved by the Animal Research Committee of the University of Fukui (Approval number: 28091) and conducted in accordance with the University of Fukui regulations on animal experiments, and the Association for Research in Vision and Ophthalmology Statement for the Use of Animals in Ophthalmic and Vision Research. The study was reported in accordance with the ARRIVE guidelines.

### *Ex vivo* assays

Rats were euthanized by CO_2_ asphyxiation and lenses were extracted. All lenses were cultured in 2 mL M199 medium containing 0.1% BSA and 30 mM galactose for 2–4 days using an incubator set at 5% CO_2_ and 37°C to induce cataracts as described previously [13]. Once opacity was induced, lenses were photographed under a microscope and same medium was replaced. At this time, the Atm inhibitors AZD0156 (MedChemExpress) or KU55933 (ChemScene), were dissolved in 16 μL DMSO per lens for final concentrations of 2.5, 5, 10, 20, and 40 μM. For the untreated lenses, vehicle control (16 μL DMSO) was added. Subsequently, lenses were cultured 2–4 days and photographed under a microscope. Control samples without cataracts were incubated for 6 days in 2 mL of M199 medium containing 0.1% BSA and galactose vehicle control (sterile water).

### Microscopic observation

Photographs of the lenses were taken in a dark room using an SZX12 stereomicroscope fitted with a DP58 camera (Olympus), as described previously [13]. Photographs were captured in a 35 mm Petri dish containing 7 mL PBS. Lens opacity was quantified using ImageJ. A weighted average was calculated from the brightness (0–255) of the area of the lens that was opacified by incubation with galactose, and the weighted average was again calculated from the brightness of the same area in the lens after addition of the inhibitor and further incubation. The change in opacity was calculated by subtracting the value after the addition of the inhibitor from the value before the addition of the inhibitor.

### Microarray data analysis

Microarray analysis was performed on control samples, galactose samples, KU55933 samples, and AZD0156 samples. A GeneChip Rat Gene 2.0 ST array chip (Thermo Fisher Scientific) was used to perform microarray experiments as described previously [31]. Preprocessing and data analyses were performed using R software. First, the data for all samples were normalized by the Robust Multi-array Average algorithm. Probes not linked to genes or genes with signal values < 5 in all samples were excluded from analyses. The signal values for each condition were normalized to the mean of the signal values of galactose-treated lenses. Genes that were significantly (*P* < 0.05) downregulated in the control and ATM inhibitor-treated samples compared to the galactose samples were selected as genes of interest. The relationships between the genes of interest genes were analyzed using STRING analysis (https://string-db.org/) [32]. Data are available under accession number GSE194074 (https://www.ncbi.nlm.nih.gov/geo/query/acc.cgi?acc=GSE194074).

### RNA extraction, cDNA preparation, and real-time RT-qPCR

RNA extraction from the lens and real-time RT-qPCR were performed as described previously [31]. Primers used are listed in S1 Table. Target gene expression levels were normalized to *Gapdh* expression levels. To evaluate differences between the galactose group and the control or drug group, a test of equal variance was performed: a two-tailed Student’s t-test was used when there was equal variance, and a two-tailed Welch’s t-test was used when there was no equal variance. *P* < 0.05 (compared with galactose) was considered statistically significant. Statistical analyses were performed using Microsoft Office Excel.

## Results

### Atm inhibition alleviates galactose-induced lens opacity

**Fig 1:**
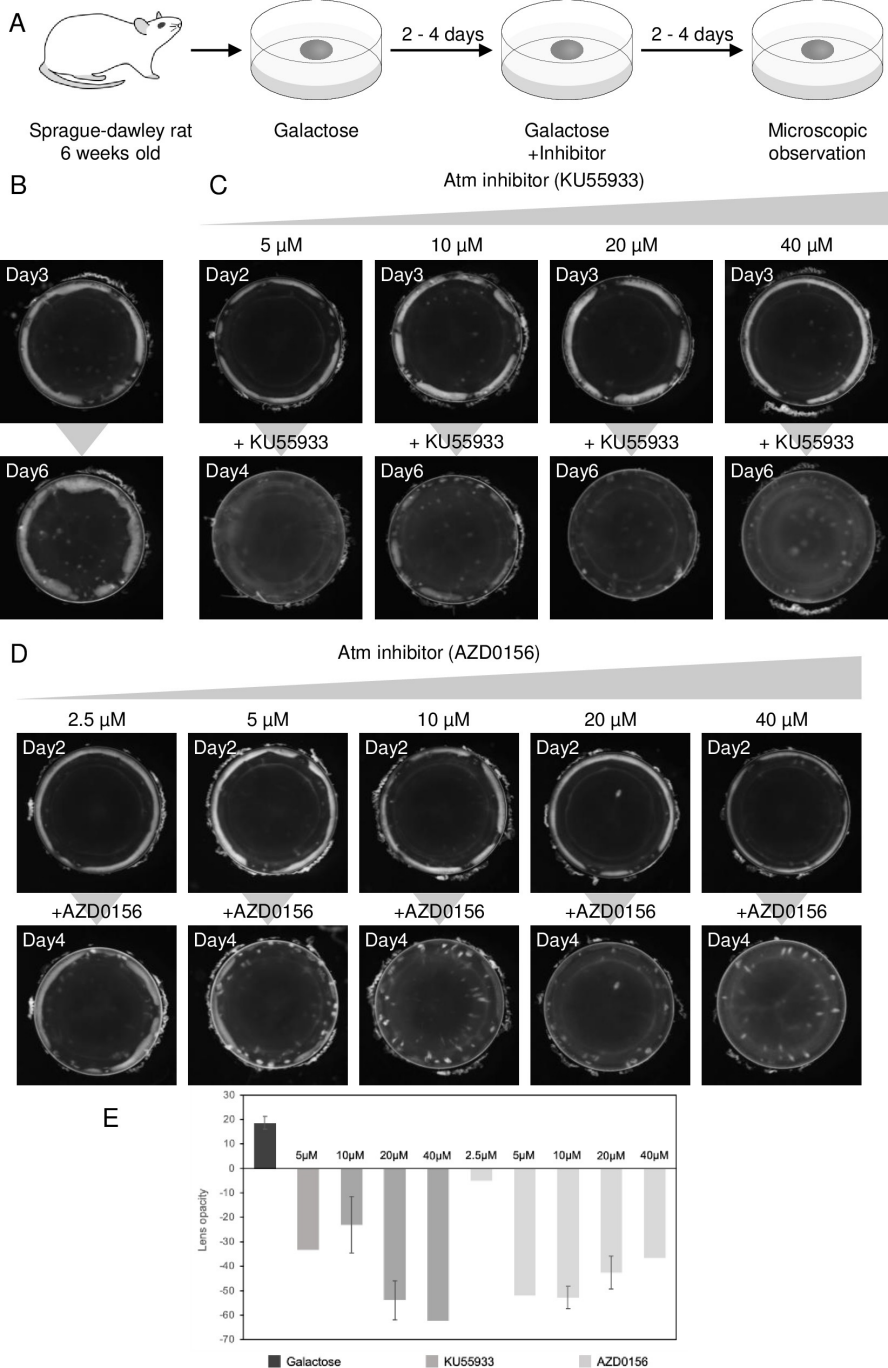
Effect of ATM inhibitors on the reduction of opacity. **(A)** Experimental scheme. **(B)** Lenses cultured in medium containing 30 mM galactose for 2–4 days (upper panel). Subsequently, DMSO vehicle control was added, and lenses were incubated in galactose-containing medium for a subsequent 2–4 days (lower panel). **(C)** Results of 2–4 days of culture in medium containing galactose as in B (upper panel) with KU55933 dissolved in DMSO to final concentrations of 5, 10, 20, and 40 μM and incubated 2–4 days (lower panel). **(D)** Results of inducing white turbidity with galactose as in C (upper panel) and a subsequent 2–4 days in galactose medium containing AZD0156 dissolved in DMSO to final concentrations of 2.5, 5, 10, 20, and 40 μM (lower panel). **(E)** We calculated the opacity region of the lens without the inhibitor, with KU55933, and with AZD0156, and then calculated the change in opacity before and after addition of the inhibitors [13]. Data are expressed as the mean ± SE. Samples used for quantification are shown in S1 Fig.

We evaluated the effect of KU55933 on opacity that had already been induced (Fig 1A). First, lenses were extracted from Sprague-Dawley rats and cultured in medium containing galactose for 2–4 days to induce opacity. As shown in Fig 1A, lenses exposed to galactose developed a ring of white opacity in the cortical equatorial region. These lenses were further cultured in galactose-containing medium with or without Atm inhibitors for 2–4 days. In lenses cultured in medium containing only galactose (Figs 1B and S1), the area of opacity further increased. By contrast, in lenses cultured with galactose and KU55933 (5, 10, 20, and 40 μM), most of the opacity was eliminated at 20 μM, and the decreases in opacity became weaker as the concentration decreased. At a high concentration (40 μM), the entire lens became slightly more opaque (Fig 1C). AZD0156, an additional Atm inhibitor, was also used at 2.5, 5, 10, 20, and 40 μM. Similar results were obtained with AZD0156 (Fig 1D). Next, we quantified the change in turbidity before and after addition of the inhibitor (Fig 1E). A photograph of the lens used for quantification is shown in S1 Fig. Photographs showing the effects of the treatment revealed pointed or banded cortical opacity in the periphery of lenses exposed to galactose, where the effect of inhibitor treatment was observed. Pointed and banded cortical opacities are early morphological features of galactose-induced cataracts [33]. As described above, the two inhibitors were found to reduce opacity in a concentration-dependent manner, and the optimal concentrations for both inhibitors were determined. In a previous study, we reported that opacity formation is also inhibited by inhibition of Plk3, a known Atm target of [13]. Therefore, we determined if GW843682X, a Plk3 inhibitor, was effective in reducing opacity once formed. Unlike the Atm inhibitors, GW843682X did not effectively alleviate opacity (S2 Fig). These results suggest that Atm inhibition, but not Plk3 inhibition, reduced galactose-induced opacity in rat lenses, suggesting that inhibition of opacity formation and reduction of previously induced opacity are regulated by different mechanisms.

### Microarray analysis of genes affected by galactose-induced opacity and Atm inhibitors

**Fig 2:**
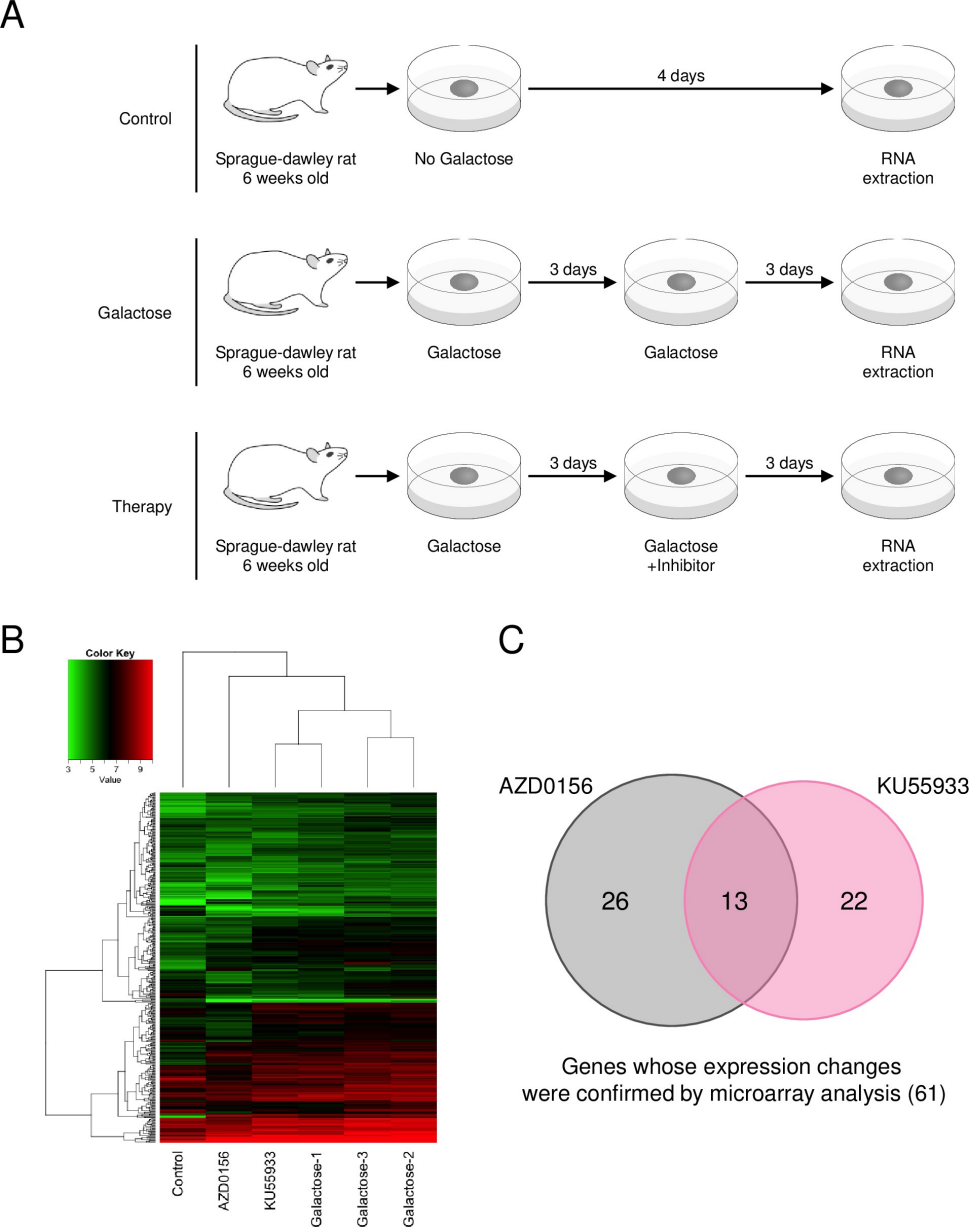
Microarray analysis of genes affected by galactose, AD0156, and KU55933. Microarray analysis was performed on lenses without opacity induction (Control), lenses with galactose-induced opacity (Galactose), and lenses treated with galactose and Atm inhibitors (KU5533 or AZD0156). **(A)** Experimental scheme. **(B)** Heatmap of gene expression in ATM inhibitor-treated samples. The red to green gradient indicates the weight of the signal value, with higher values in red and lower values in green. **(C)** Venn diagram of genes downregulated by galactose and upregulated by treatment with ATM inhibitors. The repeats for the three galactose samples were averaged to calculate the signal values for each gene. First, genes significantly (*P* < 0.05) upregulated in galactose relative to control samples were extracted (*P* < 0.05). Subsequently, genes significantly (*P* < 0.05) downregulated in the AZD0156 and KU55933 groups relative to the Galactose group were extracted. The number and relationship of genes extracted from each inhibitor are shown.

To identify genes potentially involved in the formation of galactose-induced lens opacity and reduction of opacity by Atm inhibition, we performed microarray analysis of samples cultured without galactose (Control), samples cultured in medium containing galactose (galactose), and samples cultured in medium containing galactose for 3 days to allow opacity formation and subsequently cultured with the Atm inhibitors (AZD0156 or KU55933) for 3 days (Fig 2A). Target genes were extracted after comparing the results of each analysis. In the present study, we used a 6-day galactose culture sample for analysis to determine the effect of the inhibitors on the reduction of opacity once formed. Among the 163 genes with average signal values of the three galactose samples (Triplicate) significantly increased (*P* < 0.05) compared with the signal values of the Control group. In addition, we identified 182 genes whose expression was predominantly suppressed (P<0.05) in AZD0156-treated samples, and 174 genes whose expression was predominantly suppressed (P<0.05) in KU55933-treated samples, compared with the signal values (average triplicate values) in the galactose group. Heatmaps were generated for a total of 424 genes differentially expressed in control, AZD0156, or KU55933 samples compared with galactose samples (Fig 2B). Controls were clustered at the most distant positions, and the galactose triplicates were clustered relatively close together. AZD0156 showed a different expression profile from galactose, whereas KU55933 showed an expression profile very close to that of galactose. Overall, 39 genes were downregulated significantly by AZD0156, and 35 by KU55933, compared with galactose (*P* < 0.05, S2 Table). We also identified 13 genes that were downregulated by both inhibitors (Fig 2C).

**Fig 3:**
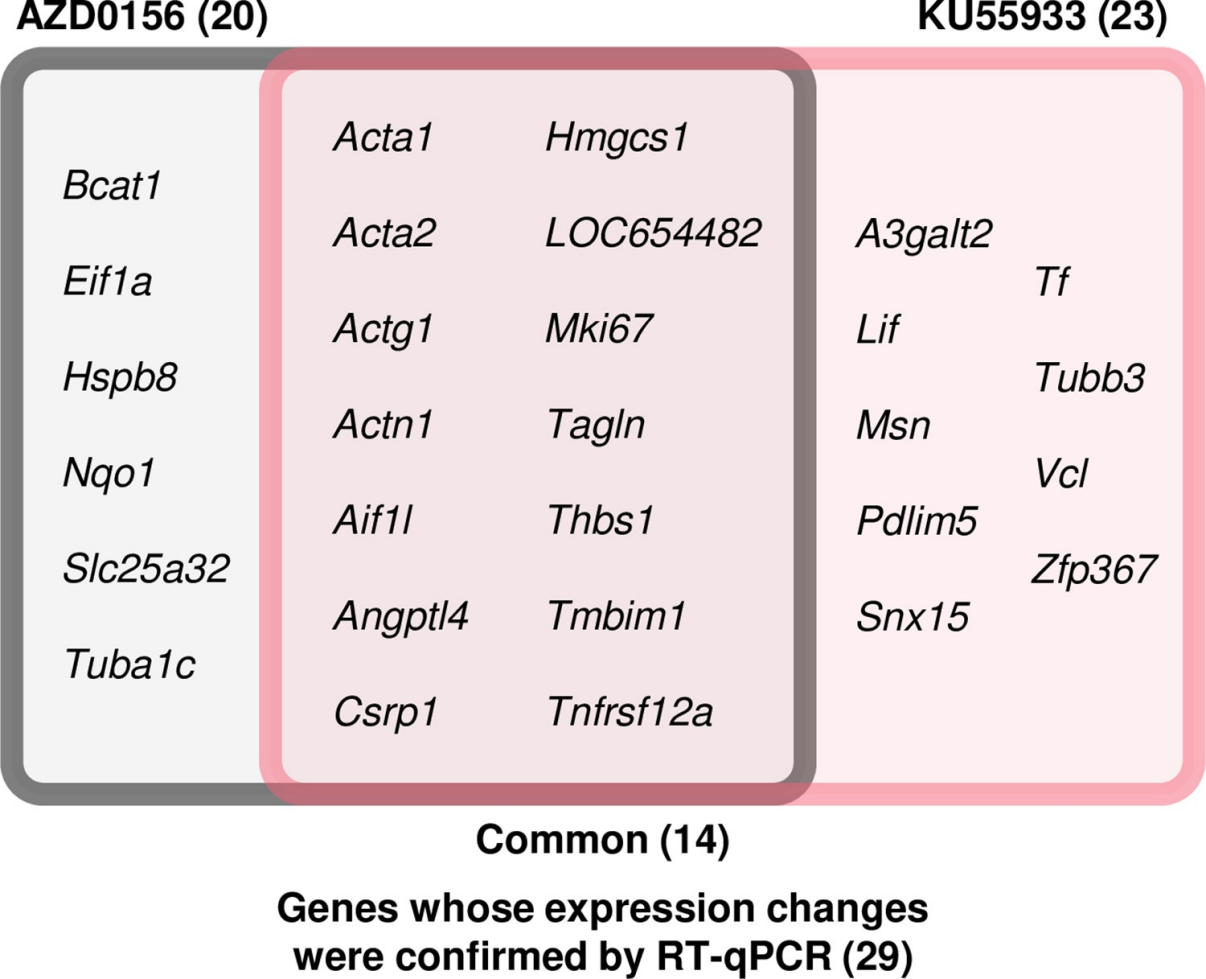
Relationship between genes affected by both Atm inhibitors. RT-qPCR was performed on genes extracted by microarray analysis. Among the genes that were significantly changed in the Control group relative to the Galactose group, the names of 20 genes that were significantly changed in the AZD0156 groups and 23 genes that were significantly changed in the KU55933 group and their relationships are shown.

**Fig 4:**
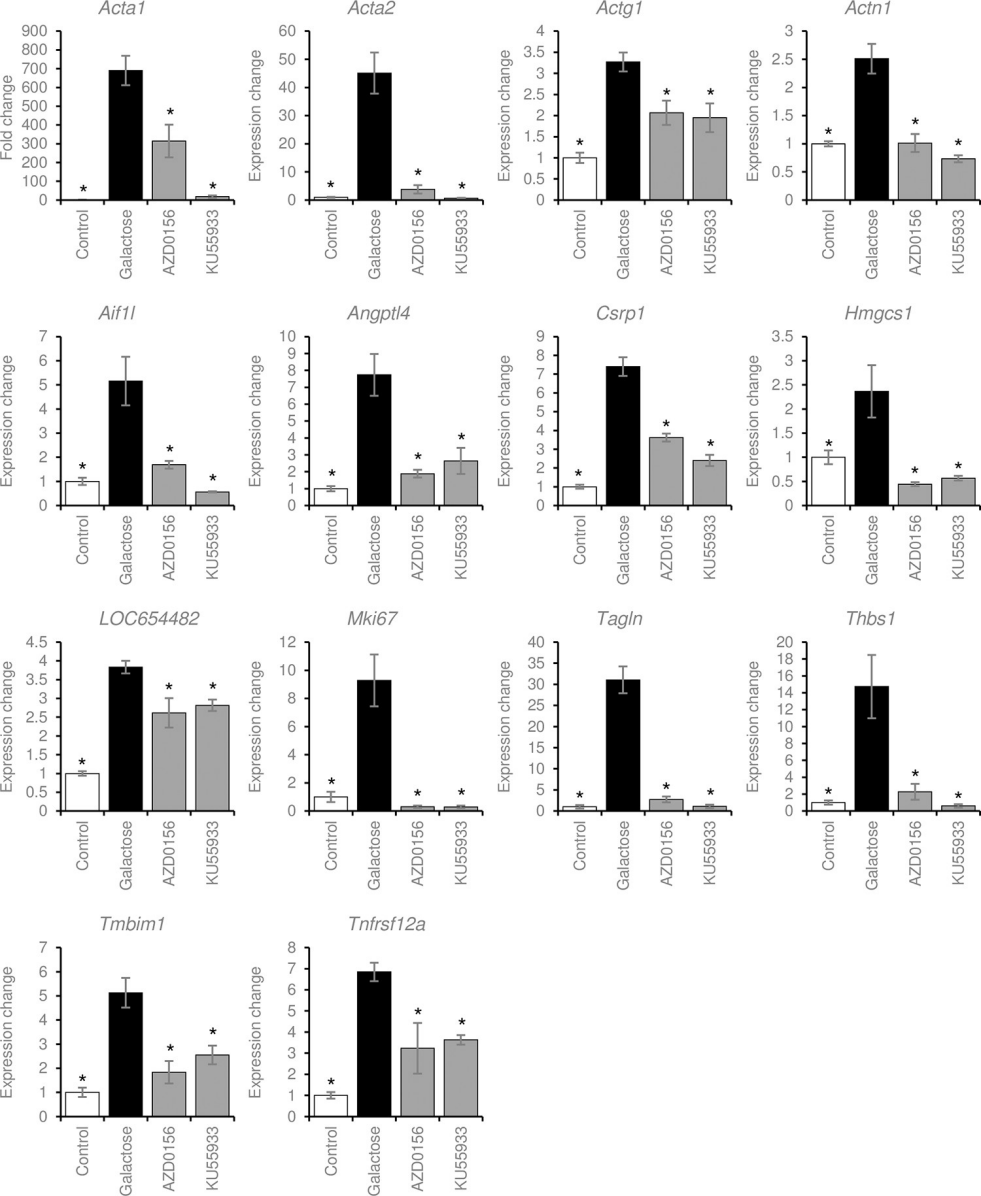
RT-qPCR results for genes with altered expression. RT-qPCR was performed on the 61 genes extracted from microarray analysis. Only the 12 genes that were significantly changed in the Control, AZD0156, and KU55933 groups relative to the Galactose group are shown. Target gene mRNA levels were normalized to *Gapdh* mRNA levels. Data are expressed as means ± SEM. *P < 0.05 relative to galactose.

RT-qPCR was performed to quantitatively measure expression of the 61 genes affected by galactose, AZD0156, or KU55933. Among the genes differentially expressed in the Control and galactose groups, 20 genes were differentially expressed in the KU55944 group relative to the galactose group, 31 genes were differentially expressed in the AZD0156 group relative to the galactose group, and 14 genes were commonly changed in both the AZD0156 and KU55933 groups (Fig 3). The RT-qPCR results for the 14 genes altered by both inhibitors are shown in Fig 4, and the RT-qPCR results for the genes that were only altered by one inhibitor are shown in S3 Fig. Genes whose expression decreased in the galactose-treated group and increased in the Atm inhibitor-treated group were analyzed in the same way, and real-time PCR was performed; however, few genes returned to control levels, and no quantitatively dominant expression changes were observed. Therefore, we focused only on genes whose expression increased in the galactose group and was suppressed in the Atm inhibitor-treated group.

### Functional analysis of genes affected by Atm inhibition

**Fig 5:**
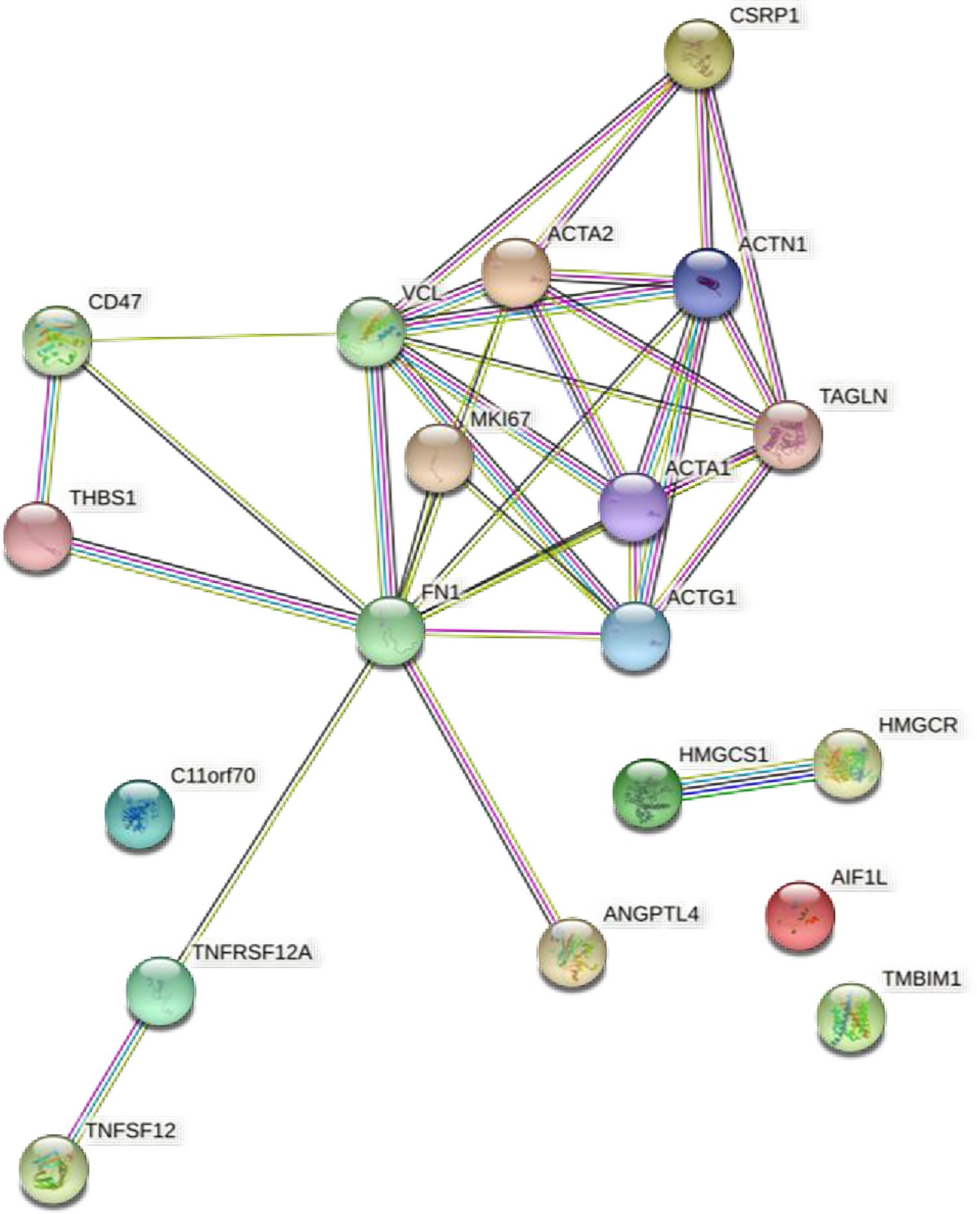
Analysis of protein interaction networks by STRING. We analyzed the protein interaction network using STRING for the 14 genes that were rescued by both inhibitors, as demonstrated by RT-qPCR. Rat gene names were converted to human gene names. As a result, rat LOC654482 was imputed as human C11orf70. From the results shown in S4 Fig, we also added more Predicted Functional Partners to the network. The color of each edge indicates the type of relationship as follows: light blue = "from curated databases"; dark purple = "experimentally determined"; green = "text mining"; black = "co-expression"; and light purple = "protein homology”.

Functional clusters for the 12 genes rescued by both drugs were investigated by analyzing protein interactions using STRING (S4 Fig). Among these genes, *Acta1*, *Acta2*, *Actg1*, *Actn1*, *Csrp1*, *Tagl*, and *Mki67* formed a functional network. Several members of the actin family, which regulates the cytoskeleton, were identified in the functional network. Acta2 is a known mesenchymal cell marker [34], and Actn1 and Tagln, actin-binding proteins connected to Acta2 in the functional network, are involved in EMT activation [35–37]. Csrp1, which was also connected to Acta2, is involved in cytoskeletal remodeling [38,39]. We postulated that inhibition of these cytoskeleton-related genes was associated with the reduction of galactose-induced opacity. Using STRING analysis to further analyze the functional relationships between the differentially altered genes, we investigated proteins that are potentially associated with the functional network (Fig 5). HMGCR, TNFSF12, CD47, VCL, and FN1 were identified as predicted partners. A large network was generated around FN1, which was associated with 11 proteins in a functional network. FN1 is a central EMT regulator [40,41]. THBS1, which was networked with FN1 and altered by Atm inhibition, promotes EMT in some cell types [42], and ANGPTL4, which had the same expression trend, regulates the increase in cellular energy required for EMT [43]. Although FN1 expression at the gene level was not altered, blocking the abnormal differentiation of lens epithelial cells by mechanisms related EMT with Atm inhibition could be important for the reduction of lens opacity. Therefore, we checked for the Fn1 gene again, and found that in addition to the low expression level in all samples, expression did not increase in the galactose samples compared with the controls; thus, Fn1was excluded from the analysis. In fact, when real-time PCR was performed, there was little change upon galactose treatment; however a decrease in expression was observed upon addition of the inhibitor (S5 Fig).

## Discussion

Diabetic cataracts are at high risk of developing even in young patients. Currently, the only treatment for cataracts is lens replacement surgery, and development of therapeutic agents is clinically desirable. Currently, the only reported drugs that can alleviate cataracts in animal models are lanosterol and sterol related compounds (VP1-001) [7,44]. These interventions enhance the chaperone activity of alpha-crystallin, thus preventing the protein aggregation related to nuclear cataracts. However, the efficacy of these drugs in humans is still unknown [45], and they might not be applicable to diabetic cataracts, which cause cortical opacity. In the present study, we determined if Atm inhibition was effective in reducing opacity using an *ex vivo* model of galactose-induced cataract. When the lenses were incubated in galactose-containing media and formed white opacities, they were further incubated with Atm inhibitors or vehicle control, revealing that the white opacity in most of the lens cortex was reduced. In addition, microarray analysis was used to extract genes that were highly expressed in galactose-induced opacity and downregulated by Atm inhibition, and could thus be related to cataract treatment.

Previous studies of the relationship between age-related cataracts and Atm have reported that UVB exposure induces DNA damage in LECs, which increases ATM activation and p53/p21 induction, leading to cell cycle arrest and senescence [46]. In our prior study, we found that Plk3 and Atm were involved in opacity formation in galactose-induced cataract, and activated apoptotic signaling [13]. This effect could be mediated by Plk3, which is upregulated by p53 [47]. These findings suggest that Atm-mediated DNA damage could contribute to the development of cataracts. In the present study, we demonstrated that the Atm inhibitors KU55933 and AZD0156 can reduce previously formed lens opacity (Fig 1). On the other hand, GW843682X, a Plk3 inhibitor, did not affect opacity (S2 Fig). Microarray analysis also revealed that among the genes upregulated by cataract and suppressed by both Atm inhibitors, no genes were directly related to apoptosis. In galactose cataracts, suppression of overactivated Atm was suggested to be important in alleviating opacity, but the downstream causal relationship between apoptosis and opacity formation should be further investigated.

Microarray analysis also demonstrated that several cytoskeleton-related genes were induced by opacity formation and repressed by Atm inhibitors (Figs 5 and S5). Actin alpha 2, smooth muscle (Acta2) encodes α-smooth muscle actin (α-SMA), which is typically expressed in vascular smooth muscle cells [48]. In the lens, α-SMA, a known marker of mesenchymal cells, is upregulated in mesenchymal cells resulting from LEC EMT [34]. A prior study demonstrated that cataracts induced by high glucose strongly express α-SMA, and that quercetin inhibits cataracts by reducing α-SMA expression and suppressing EMT [23]. In addition, LECs in human diabetic cataracts express higher levels of α-SMA than LECs in age-related cataracts, suggesting possible involvement of EMT in diabetic cataracts [24].

Decreased expression of the mesenchymal cell marker Acta2 by Atm inhibition could be an important indicator of turbidity reduction. ACTN1 encodes α-actinin, an actin crosslinking protein involved in cell adhesion [49]. Overexpression of ACTN1 in certain cancer cells induces EMT, and inhibition of Actn1 by Oroxylin A decreases expression of αSMA and suppresses EMT. [35,50]. Transgelin (Tagln), an actin-binding protein, also promotes EMT in cancer cells [36,37], and expression changes of these genes could be indicative of cytoskeletal remodeling. Analysis of the protein interaction network by STRING, including predicted related factors, revealed a network centered on FN1 and connected to ACTA1, ACTA2, ACTG1, ACTN1, ANGPTL4, MKI67, TAGLN, THBS1, and TNFRSF12A, which were downregulated by Atm inhibition (Fig 5). Fibronectin (FN1) is involved in cell adhesion and cell differentiation via cellular interaction with the extracellular matrix [40]. Administration of fibronectin to lens epithelial cells triggers EMT and induces expression of α-SMA [41]. In addition, THBS1, which is connected to FN1, is associated with EMT activation, and knockdown of THBS1 suppresses α-SMA expression [42,51]. In addition, the connected ANGPTL4 stabilizes proteins important for EMT by regulating cellular metabolic activity [43]. Expression of the Fn1 gene changed little after galactose treatment, but was suppressed by Atm inhibitors, suggesting that it may be involved in Atm-regulated EMT. When EMT is inducted in LECs, α-SMA is upregulated and LECs transform to fibroblast-like cells, compromising their differentiation into normal lens fiber cells [52]. These results suggest that galactose-induced EMT in LECs is one of the causes of opacity, and that blockade of this axis with Atm inhibition normalizes cell differentiation, decreasing opacity.

Activation of Atm kinase is associated with Tip60 acetyltransferase [53]. Tip60-mediated Atm acetylation results in Atm phosphorylation, and the activated Atm-Tip60 complex activates Atm the target protein. Therefore, blocking Tip60 decreases Atm activation [54]. In our unpublished findings, we have identified that the Tip60 inhibitor TH1834 decreases galactose-induced lens opacity in rats [unpublished data]. EMT-related genes such as Acta2 and Tagln were also downregulated by TH1834. These effects could be the result of blocking the Tip60/Atm axis.

Gene expression analysis did not identify differentially regulated genes directly related to DNA damage. Atm and downstream signaling pathways are primarily activated by phosphorylation of target proteins [27], and microarray-based analysis of gene expression changes would not reflect changes in protein phosphorylation. Further validation at the protein level is needed to elucidate these mechanisms in detail.

In summary, we demonstrated in the present study that Atm inhibition reduced galactose-induced lens opacity. Atm inhibition could have eliminated previously formed opacity by suppressing expression of genes related to the cytoskeleton and EMT. We have thus identified a new regulator of EMT in the pathogenesis of diabetic cataracts based on gene expression changes. Atm inhibitors and drug therapies targeting these downstream factors could provide insight into new cataract therapies.

## Supporting information

S1 Fig Effect of ATM inhibitors on the galactose-induced cataracts used in this study. The upper part of the photograph shows an image taken before addition of the inhibitor, and the lower part shows an image taken after addition of the inhibitor. In the photograph, “q” on the left denotes the sample used for qRT-PCR, and “M” denotes the sample used for microarray analysis.
(PDF)

S2 Fig The Plk3 inhibitor GW843682X does not affect galactose-induced opacity. Results of 4-day incubation in medium containing galactose (left panel) and 4-day incubation in medium containing galactose with GW843682X dissolved in DMSO to a final concentration of 10 μM (Right panel).
(PDF)

S3 Fig RT-qPCR analysis of genes with altered expression in microarray analysis. RT-qPCR was used to measure expression of 61 genes extracted by microarray analysis. Among the genes significantly altered in the Galactose group relative to Control, the genes altered only by AZD0156 **(A)** and the genes altered by only KU55933 **(B)** are shown. Results are expressed as target gene mRNA levels normalized to *Gapdh* mRNA levels. Data are expressed as mean ± SEM. *P < 0.05 relative to galactose.
(PDF)

S4 Fig STRING analysis of protein interaction networks. We analyzed the protein interaction network using STRING for 14 genes that were altered by both inhibitors, as demonstrated by RT-qPCR. This is the preliminary stage of the analysis shown in Fig 5.
(PDF)

S5 Fig Measurement of Fn1 expression by RT-qPCR. Results are shown as target gene mRNA levels normalized to Gapdh mRNA levels. Data are as the mean ± SEM. *P < 0.05, relative to galactose.
(PDF)

S1 Table List of primers used for RT-qPCR. (XLSX)

S2 Table List of genes with altered expression extracted from microarray analysis. Genes differentially regulated by either of the two Atm inhibitors shown in Fig 2 are indicated by “◆”. Lines 4–9 show the signal values for each sample, lines 10–12 show the signal values for the control and inhibitor samples minus the average of the three galactose signal values, and lines 13–15 show the *P* values.
(XLSX)
