# Expression of Concern: Atm inhibition decreases lens opacity in a rat model of galactose-induced cataract

**DOI:** 10.1371/journal.pone.0329284

**Published:** 2025-07-31

**Authors:** The PLOS One Editors

After this article [1] was published, concerns were raised about overlapping samples and microarray datasets between this article and articles [2–6] with the same ethical approval number. Specifically:

- The Control sample overlaps with articles [2–6].
- The three Galactose samples overlap with articles [2,4], and [6].

In response to queries about these concerns, the corresponding author stated that each article [1–6] is interconnected and used data from the same control and galactose microarrays in order to compare samples collected at different time points, whilst the inhibitor samples in each article differ, and that [1] is a follow-up to [4]. They stated that Robust Multichip Analysis correction was carried out for each raw dataset, and that differences arose in the processed data due to the different inhibitors in each article.

The corresponding author stated that [1] was carried out with the aim of contributing to the elucidation of the full mechanism of cataract development by using Atm in [1] and other inhibitors in [2–4] and [6] to identify genes associated with cataract development.

Based on input received from the *PLOS One* Editorial Board and a statistical reviewer, PLOS concluded that the microarray study design and data analyses were not performed to a high technical standard and call into question the reliability of the microarray results. Specifically:

- The microarray experiments did not include sufficient replication and only one control sample was included in the microarray study.
- The different experimental groups for microarray experiments were not matched for number of days in culture.
- The control conditions were not well-matched to experimental conditions in some cases.
- The reuse of control and galactose-treated samples across articles [1–6] introduces statistical dependency which inflates Type I error rates, and article [1] did not report statistical analyses or adjustments needed to address this issue.
- Treating shared samples as independent and relying on technical replicates artificially increases the apparent sample size, leading to underestimated variability and overstated statistical significance.
- Microarray data were not obtained in a single batch/experiment.

The corresponding author provided additional information about the study design, specifically, that for RT-qPCR, n = 3 or more, of which 2 were obtained from two different animals (one eye = control and one eye = experimental), and the third was the RNA sample used for the microarray. They also stated that experimental groups were defined based on having the same opacity condition, rather than by time in culture.

Based on input from the *PLOS One* Editorial Board, PLOS concluded that the reliability of the microarray data is in question given the study design concerns, but the main conclusions in [1] are supported by the RT-qPCR results which are not critically dependent on the microarray results and which validated the microarray findings pursued in [1] for follow-up studies.

The RT-qPCR data for [1] is provided here and the Data Availability statement is updated to: Microarray data are available in the GEO repository under the accession number GSE194074 (https://www.ncbi.nlm.nih.gov/geo/query/acc.cgi?acc=GSE194074). RT-qPCR data are available in the Supporting information files.

The *PLOS One* Editors issue this Expression of Concern to inform readers of the above study design concerns and to provide the RT-qPCR data for [1].

## Supporting information

S1 File Underlying RT-qPCR data for [1]. (XLSX)

## References

[1] Nagaya M, Kanada F, Takashima M, Takamura Y, Inatani M, Oki M. Atm inhibition decreases lens opacity in a rat model of galactose-induced cataract. PLoS One. 2022;17(9):e0274735. doi: 10.1371/journal.pone.0274735 36149903 PMC9506662

[2] Takashima M, Nagaya M, Takamura Y, Inatani M, Oki M. HIF-1 inhibition reverses opacity in a rat model of galactose-induced cataract. PLoS One. 2024;19(2):e0299145. doi: 10.1371/journal.pone.0299145 38416732 PMC10901314

[3] Kanada F, Takamura Y, Miyake S, Kamata K, Inami M, Inatani M, et al. Histone acetyltransferase and Polo-like kinase 3 inhibitors prevent rat galactose-induced cataract. Sci Rep. 2019;9(1):20085. doi: 10.1038/s41598-019-56414-x 31882756 PMC6934598

[4] Nagaya M, Yamaoka R, Kanada F, Sawa T, Takashima M, Takamura Y, et al. Histone acetyltransferase inhibition reverses opacity in rat galactose-induced cataract. PLoS One. 2022;17(11):e0273868. doi: 10.1371/journal.pone.0273868 36417410 PMC9683626

[5] Yamaoka R, Kanada F, Nagaya M, Takashima M, Takamura Y, Inatani M, et al. Analysis of cataract-regulated genes using chemical DNA damage induction in a rat ex vivo model. PLoS One. 2022;17(12):e0273456. doi: 10.1371/journal.pone.0273456 36477544 PMC9728860

[6] Masuda F, Inami M, Takamura Y, Inatani M, Oki M. Identification of genes contributing to attenuation of rat model of galactose-induced cataract by pyruvate. Genes Cells. 2024;29(10):876–88. doi: 10.1111/gtc.13150 39219252 PMC11555625

