# Supplementary figures and images for: Atm inhibition decreases lens opacity in a rat model of galactose-induced cataract
Source: PLoS One. 2022 Sep 23;17(9):e0274735. doi: 10.1371/journal.pone.0274735 (PMC9506662; doi:10.1371/journal.pone.0274735)

## Galactose (n=5)

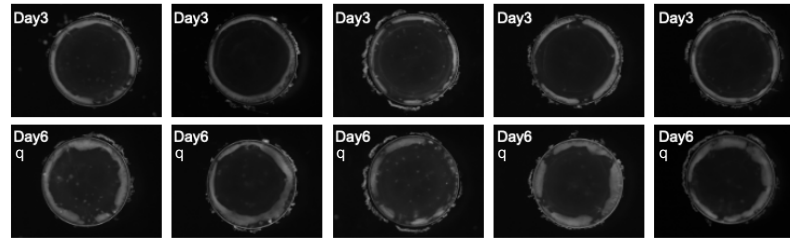

## KU55933

5 $\mu$ M

10 $\mu$ M (n=3)

20 $\mu$ M (n=6)

40 $\mu$ M

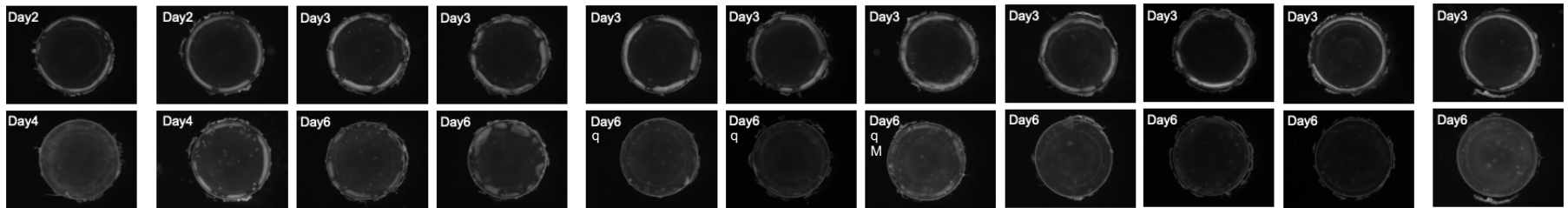

## AZD0156

2.5 $\mu$ M

5 $\mu$ M (n=2)

10 $\mu$ M (n=5)

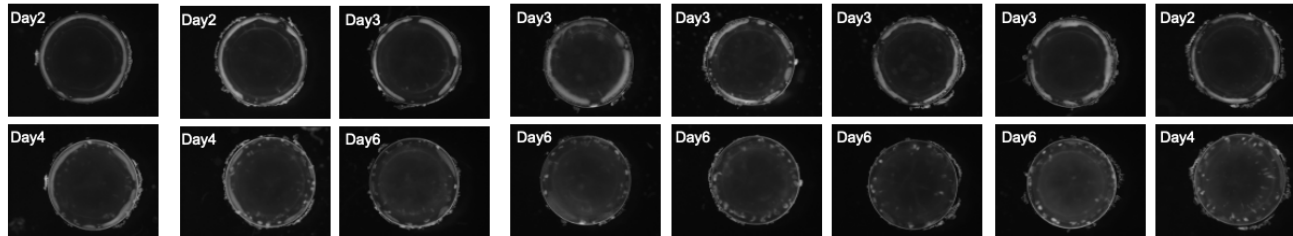

20 $\mu$ M (n=6)

40 $\mu$ M

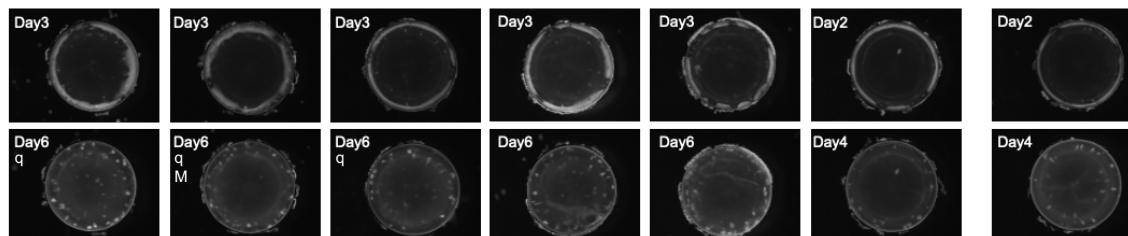

Supplement: S1 Fig — The upper part of the photograph shows an image taken before addition of the inhibitor, and the lower part shows an image taken after addition of the inhibitor. In the photograph, “q” on the left denotes the sample used for qRT-PCR, and “M” denotes the sample used for microarray analysis. (PDF) [file pone.0274735.s001.pdf]

## Plk3 inhibitor (GW843682X)

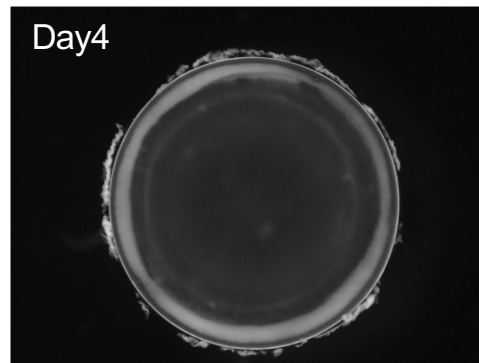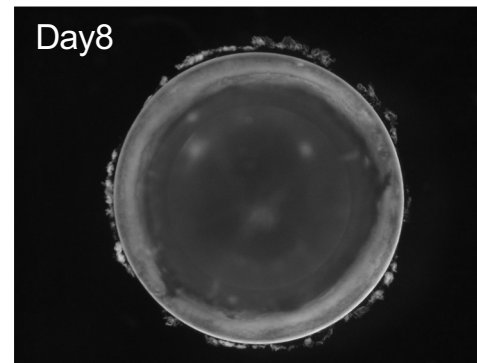

Supplement: S2 Fig — Results of 4-day incubation in medium containing galactose (left panel) and 4-day incubation in medium containing galactose with GW843682X dissolved in DMSO to a final concentration of 10 μM (Right panel). (PDF) [file pone.0274735.s002.pdf]

A

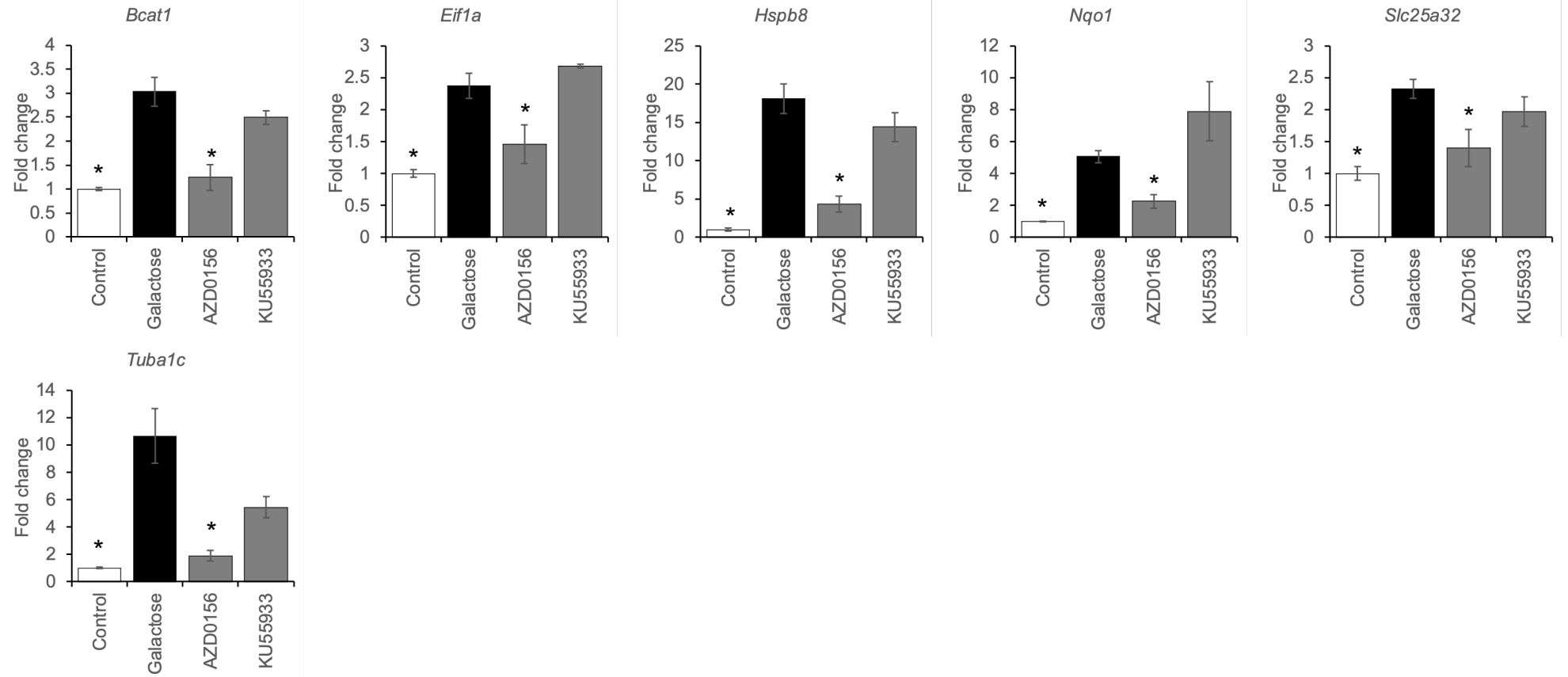

B

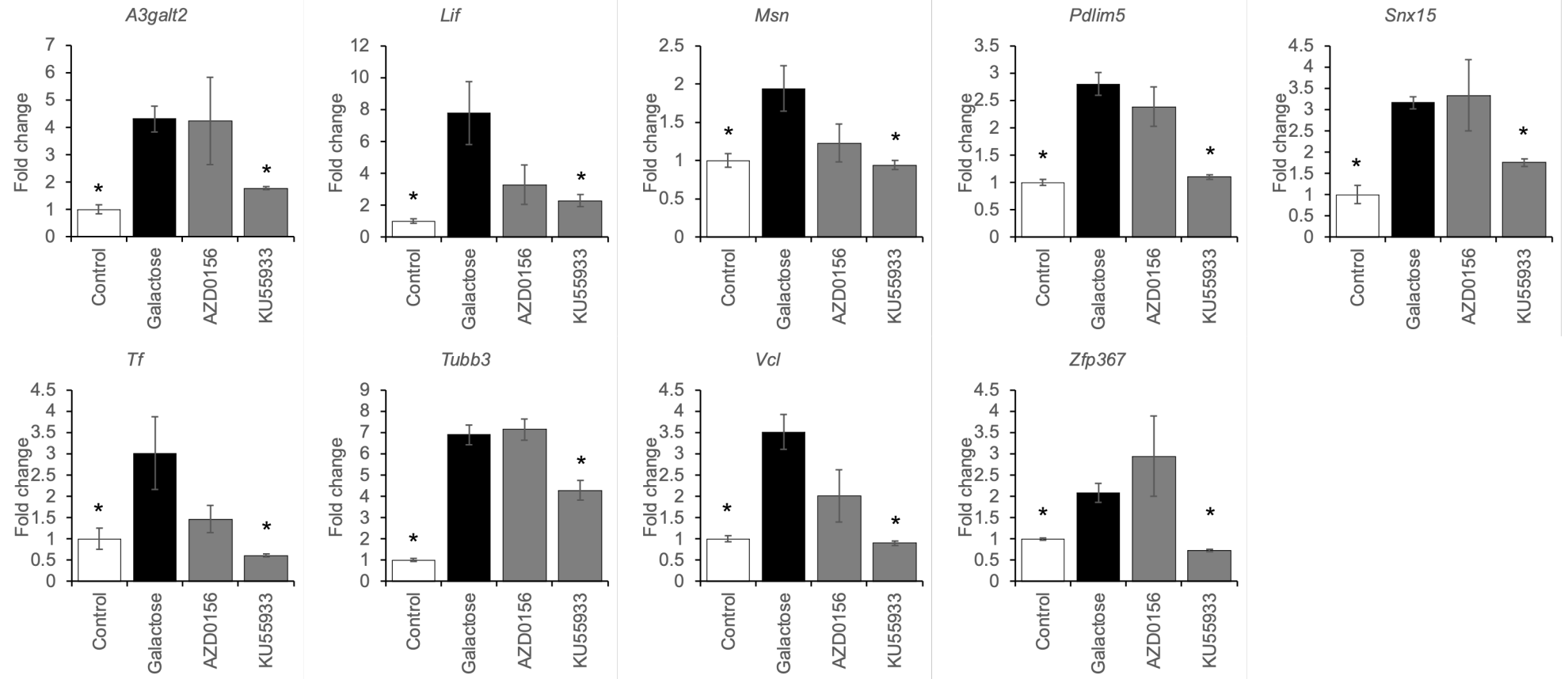

Supplement: S3 Fig — RT-qPCR was used to measure expression of 61 genes extracted by microarray analysis. Among the genes significantly altered in the Galactose group relative to Control, the genes altered only by AZD0156 (A) and the genes altered by only KU55933 (B) are shown. Results are expressed as target gene mRNA levels normalized to Gapdh mRNA levels. Data are expressed as mean ± SEM. *P < 0.05 relative to galactose. (PDF) [file pone.0274735.s003.pdf]

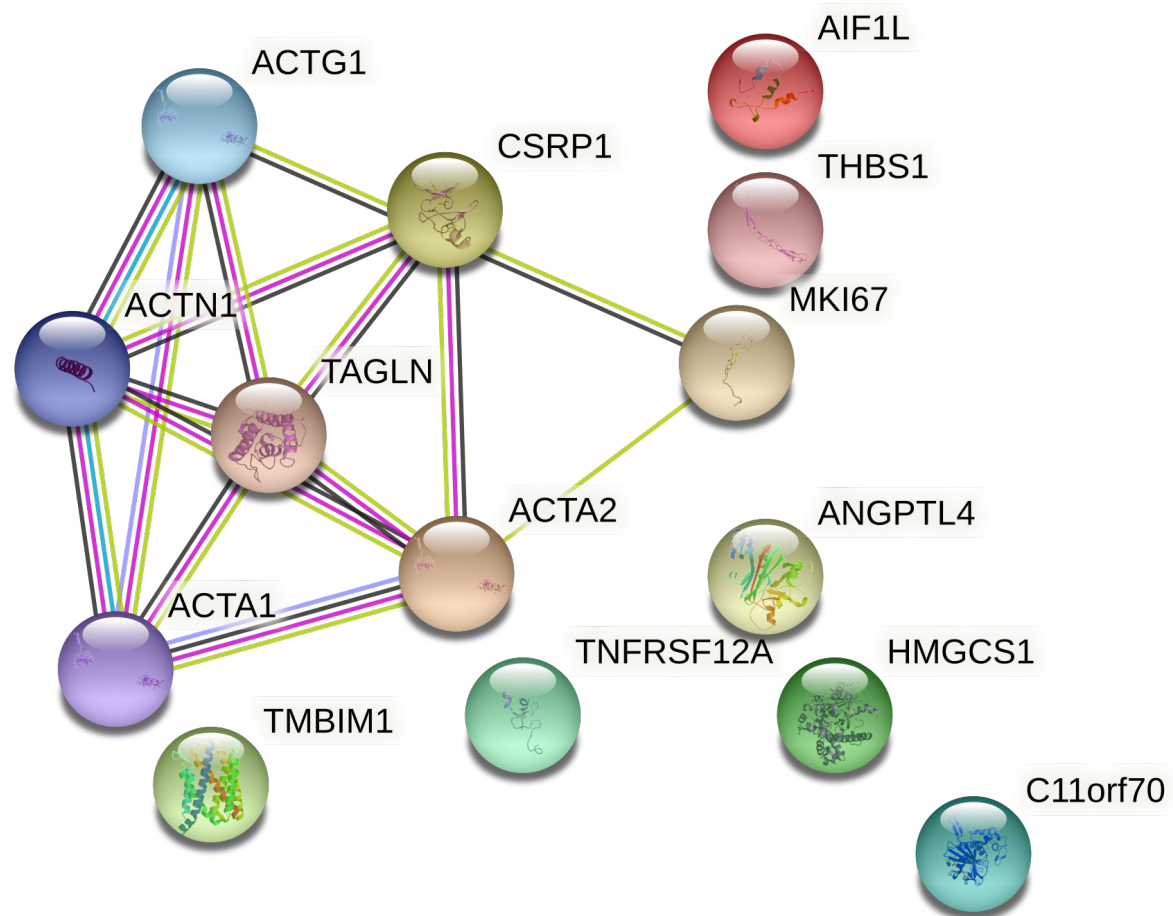

Supplement: S4 Fig — We analyzed the protein interaction network using STRING for 14 genes that were altered by both inhibitors, as demonstrated by RT-qPCR. This is the preliminary stage of the analysis shown in Fig 5. (PDF) [file pone.0274735.s004.pdf]

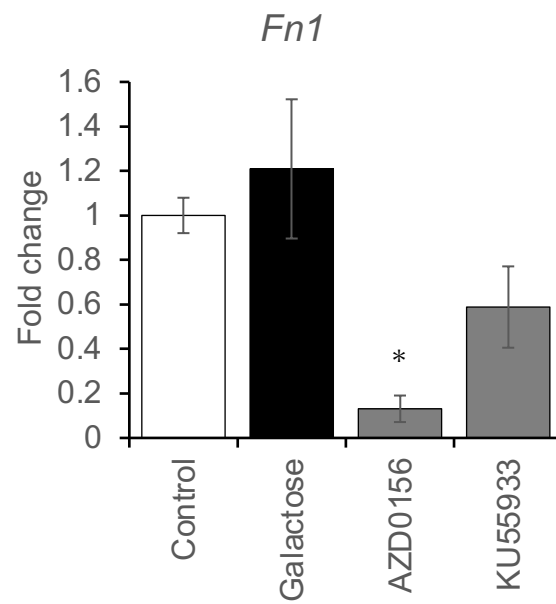

Supplement: S5 Fig — Results are shown as target gene mRNA levels normalized to Gapdh mRNA levels. Data are as the mean ± SEM. *P < 0.05, relative to galactose. (PDF) [file pone.0274735.s005.pdf]
